# High blood galectin-3 level associated with risk of frailty in aging

**DOI:** 10.3389/fendo.2023.1189192

**Published:** 2023-09-25

**Authors:** Xueying Ji, Zhaoshun Jiang, Yixuan Qiu, Jiaming Yu, Yan Zhang, Jiaofeng Wang, Bo Ye, Yuxin Huang, Weidong Gu, Yiqin Huang, Jie Chen, Zhijun Bao

**Affiliations:** ^1^ Department of General Practice, Huadong Hospital Affiliated to Fudan University, Shanghai, China; ^2^ Shanghai Key Laboratory of Clinical Geriatric Medicine, Huadong Hospital, Shanghai, China; ^3^ Department of Anesthesiology, Huadong Hospital Affiliated to Fudan University, Shanghai, China; ^4^ Department of Gastroenterology, Guangdong Academy of Medical Sciences, Guangdong Provincial People’s Hospital, Guangdong, China; ^5^ Department of Endocrinology, Huadong Hospital Affiliated to Fudan University, Shanghai, China; ^6^ Department of Geriatric Medicine, Huadong Hospital Affiliated to Fudan University, Shanghai, China; ^7^ Department of National Clinical Research Center for Ageing and Medicine, Huashan Hospital Affiliated to Fudan University, Shanghai, China

**Keywords:** aging, frailty, humoral, galectin-3, transcriptome, frail mice

## Abstract

**Background:**

Frailty is one of the most problematic expressions of population aging, but its underlying mechanism has not been fully elucidated. Circulating galectin-3 (Gal-3) is involved in the pathogenesis of many age-related diseases. This study aims to explore the influence of circulating Gal-3 on the regulation of frailty and aging and to identify the potential mechanism further.

**Methods:**

In this cross-sectional analysis, the Fried frailty phenotype (FP) was assessed among 149 community elderly residents in Shanghai. Peripheral blood mononuclear cells (PBMCs) were isolated by the Ficoll-Paque density gradient method, and differentially expressed genes (DEGs) encoding transcription factors in frailty were detected by Illumina and bioinformatics analyzed with R software. Gene Ontology (GO) enrichment analyses and Kyoto Encyclopedia of Genes and Genomes (KEGG) pathway analyses were performed to explore the functional roles of these DEGs and the target genes related to frailty phenotypes. The serum Gal-3 concentration was tested by enzyme-linked immunosorbent assay (ELISA). Mouse frailty phenotype was used to construct an *in vivo* model of frailty, after which the serum levels of circulating Gal-3 and its gene expression levels in mouse tissues were determined.

**Results:**

Participants’ mean age was 72.04 ± 7.05 years. In total, 21.48% were frail and 36.91% were pre-frail. The mean serum Gal-3 concentration was 46.34 ± 17.99 ng/mL in frail participants, 32.30 ± 8.14 ng/mL in pre-frail participants, and 26.00 ± 5.87 ng/mL in non-frail individuals (*p* < 0.001). Significant positive correlations between serum Gal-3 level and FP score, SARC-F score, C-reactive protein (CRP), interleukin-6, etc., were observed. In addition, the KEGG pathway and GO enrichment analyses showed that 265 DEGs in PBMCs of frail participants were mainly related to inflammatory response, translation, RNA binding, protein binding, ribosome, and primary immunodeficiency. *LGALS3* was identified as the overlapping gene between frailty-related DEGs and aging-related DEGs. The elevated serum Gal-3 concentration in the *in vivo* model of frailty was consistent with the results in participants.

**Conclusion:**

In both community-dwelling older adults and aged mice, serum Gal-3 concentration was positively correlated with frailty. This circulating mediator may be a promising indicator of frailty.

**Clinical trial registration:**

Chinese Clinical Trial Registry identifier, ChiCTR2000036399.

## 1 Introduction

Life expectancy is increasing globally. According to the World Health Organization, approximately 16% of the world’s population will be 65 years or older by 2050 (1, 2). Population aging is accompanied by an increased prevalence of geriatric syndromes, disability, and comorbidity. Frailty is one of the most important geriatric syndromes, which is characterized by declined physiological reserve and resilience to stressors across multiple physiological systems, and its prevalence is aggravated by age (3). Frailty can increase the risk of adverse health outcomes (4), such as falls, hospitalizations, and mortality. However, early detection and treatment could ameliorate or even potentially reverse the progression of physical frailty (5). Therefore, early identification of older adults with frailty plays an important role in monitoring health conditions, implementing health management plans, and assessing prognosis.

Galectin-3 (Gal-3, also known as Mac-2), a β-galactoside binding lectin, is widely expressed in human tissues, including all types of immune cells, epithelial cells, endothelial cells, stem cells, and sensory neurons. Furthermore, it is highly expressed and secreted by macrophages (6, 7). As a specific regulator of many biological systems, Gal-3 is highly promiscuous and localized within the tissue micro-environment, including extracellular, cytoplasmic, and nuclear (8). The different locations of Gal−3 contribute to its various functions. Gal-3 is a nucleocytoplasmic protein synthesized without a classical signal sequence, although it can be secreted through unconventional pathways (9–11). Secreted Gal-3 is pivotal in numerous biological activities including cell growth, differentiation, transformation, apoptosis, angiogenesis, inflammation, fibrosis, and host defense (12–18). Also, it is able to cross-link surface glycoproteins and stimulate important pathways involved in the innate immune response such as the oxidative burst in neutrophils, alternative macrophage (M2) activation, and mast-cell degranulation (17). Previous studies have reported that elevated blood Gal-3 level in humans was related to exacerbating disease in inflammatory, metabolic, and malignant diseases (19–23). Elevated serum Gal−3 levels have been detected in almost all types of cardiovascular disease (24). However, little is known about the function of Gal-3 in frailty. Moreover, the physiological relevance of whole-blood Gal-3 to predict aging-associated conditions clearly needs further investigation. Thus, monitoring circulating Gal-3 levels in humans could help us understand the mechanism of aging and frailty, leading the way to finding potential treatments.

A chronic state of low-grade inflammation, accompanied by a dysregulation of the inflammatory cytokine network, has been indicated as a major driver of age-associated conditions (25–27). A previous study also proposed that the association of inflammatory factors such as C-reactive protein (CRP), interleukin (IL)-6, tumor necrosis factor (TNF)-α, and IL-10 levels with frailty may reflect an endophenotypic expression of inflamm-aging (28). Hence, we speculated that the association of frailty and Gal-3 might also be mediated by inflammatory cytokine networks. Nevertheless, up to now, there is very limited evidence on the frailty status in human Gal-3-related studies. In this study, we aimed to address the change of Gal-3 levels in human whole blood with frailty. We performed serum biochemical and peripheral blood mononuclear cells (PBMC) microarray analyses in humans to determine the secretory phenotype characteristics of frailty. Furthermore, we used the frail mouse model to study the significantly altered behavioral phenotype and associated secreted Gal-3 levels in blood samples to reveal the Gal-3-dependent inflammatory dysregulation of frailty. Together, we screened sensitive biomarkers from blood samples to provide potential targets for the assessment and treatment of frailty.

## 2 Materials and methods

### 2.1 Baseline data collection

For this cross-sectional study, we recruited 149 older adults living in the Shanghai community from March to June 2021. The trial was registered on the Chinese Clinical Trial Registry with the identification number ChiCTR2000036399. The principal investigator was Zhijun Bao, and the date of registration was August 22, 2020. All participants were aged over 60 years and were in stable condition. We excluded participants with acute disease, current surgery, severe cognitive problems, or communication difficulties. All participants underwent the following procedures: the baseline data included participants’ demographics, medical conditions, frailty assessment, and other relevant factors. 1) Collection of identification and sociodemographic data regarding age, education, comorbidities (Charlson Comorbidity Index (CCI)), sarcopenia risk (SARC-F), nutrition condition (Mini-Nutritional Assessment (MNA)), and self-care ability (Barthel index). 2) Frailty assessment: frailty phenotype score was measured based on the definition by Fried et al. (2001) (29), including five characteristics: weight loss, exhaustion, low physical activity, slowness, and low grip strength; weight; height; body mass index (BMI); appendicular skeletal muscle mass (ASM); and relative ASM (RASM). The ASM mass was measured as the sum of the lean mass of both the arms and legs, and the height-adjusted RASM mass was also calculated (ASM/height^2^, kg/m^2^). 3) Blood samples were collected from the large antecubital veins after 12-h overnight fasting. All blood samples were collected into vacuum tubes containing ethylene diamine tetra acetic acid (EDTA) or serum separator tube, and Gal-3 levels were determined by the enzyme-linked immunosorbent assay (ELISA) analysis in the laboratories. All participants signed the informed consent form. The project was approved by the Research Ethics Committee under protocol No. 2019K097.

### 2.2 PBMC isolation and microarray analysis

The experiment contained 18 samples consisting of elderly participants with frailty, elderly participants without frailty, and robust young adults (N = 6, respectively). PBMCs were isolated from whole blood by density gradient centrifugation with Ficoll-Paque (17-1440-03, GE, Pittsburgh, PA, USA). Total RNA was extracted from PBMCs using TRIzol (Thermo Fisher Scientific Inc., Waltham, MA, USA). RNA integrity was assessed using the RNA Nano 6000 Assay Kit of the Bioanalyzer 2100 system (Agilent Technologies, Santa Clara, CA, USA). The clustering of the index-coded samples was performed on a cBot Cluster Generation System using TruSeq PE Cluster Kit v3-cBot-HS (Illumina, San Diego, CA, USA) according to the manufacturer’s instructions. HTSeq v0.6.0 was used to count the read numbers mapped to each gene. Differential expression analysis was performed using the DESeq2 R package (1.10.1) and the edgeR R package (3.12.1). The Limma package of R software was used to identify the differentially expressed genes (DEGs) from the young, non-frail, and frail groups. *p*-Value <0.05 and |log2 fold change (FC)| > 0.5 were regarded as the threshold to determine the DEGs (23). The Ggplot2 and Venn Diagram packages of R were applied to generate volcano plots and Venn diagrams, respectively (14). The examinations of both young adults *vs.* non-frail elderlies and non-frail *vs.* frail elderlies were performed to obtain overlapping DEGs and subsequently identify the genes unique to age-related frailty.

### 2.3 Gene Ontology and pathway enrichment analysis

The functions of the candidate DEGs were annotated using the DAVID gene annotation tool (https://david-d.ncifcrf.gov/) (30). The Gene Ontology (GO) enrichment annotations were evaluated for three sub-ontologies: biological process (BP), molecular function (MF), and cellular component (CC). In the GO enrichment analysis, the number of nodes indicates the degree of gene enrichment, and each GO item has a *p*-value <0.05. The pathway enrichment analysis was performed using the Kyoto Encyclopedia of Genes and Genomes (KEGG) (31) database. KEGG enrichment of DEGs used the cutoff criteria of *p*-value <0.05. The bubble color represents the *p*-value in enriched pathways, and the size of the bubble represents the gene number.

### 2.4 Animal experiments

The current study utilized male C57BL/6 mice at 20 weeks and 22 months of age that were obtained from Shanghai Model Organisms Center (Shanghai, China). All mice were housed on a 12-h light/dark cycle and kept at 23°C ± 1°C for 1 month at the Shanghai Laboratory Animal Research Center (Shanghai, China). Animals were fed a regular mouse diet. After 30 days, performance testing was implemented, which included body weight, body composition analysis, and physical function assessment. After 4-h fasting (from 08:00 AM to 12:00 AM), the body composition (fat and lean mass) of mice was analyzed using an animal body composition analyzer (EchoMRI, Houston, TX, USA). All the mice were carefully dissected after euthanasia with 10% chloral hydrate. Blood samples were gained from the eyeballs of mice, collected into tubes, and centrifuged for 30 min. Supernatants (serum) were collected, snap-frozen in liquid nitrogen, and stored at −80°C for further analysis. All animal studies were reviewed and approved by the ethics committees of the School of Life Science of Fudan University (Approval No. 202101004S).

### 2.5 Mouse frailty criteria

According to previous studies (32), a modified frailty phenotype for rodents included the following physical components: declined body weight (more than 10% during 1 month), walking speed (rotarod speed), strength (grip strength), endurance (time to fatigue on treadmill), and physical activity (voluntary wheel running distance). Following the percentiles used by Fried et al., these criteria were used to identify frailty cutoff values at 23 months of age (32, 33). Mice with three or more positive frailty markers were identified as frail, mice with one or two positive markers were considered pre-frail, and mice with no positive frailty marker were considered non-frail.

### 2.6 Mouse physical function assessment

The grip strength was measured three times for each mouse using a rodent grip strength meter. Each mouse performed three trials of grip strength measurement with a 10-min rest period between each trial. Strength was evaluated using a grip meter test (YLS-13A, Jinan Yiyan Company, Jinan, China). The best score of three trials was used to represent the grip strength of each mouse. The assessment of walking speed (rotarod speed), endurance (time to fatigue on treadmill), and physical activity (voluntary wheel running distance) was performed as previously described (32). Walking speed was recorded in seconds when the mouse was unable to sustain the rotation speed and fell from a rotarod (Rota-Rod R/S; LSi Letica, Cornella, Spain). Each mouse performed three trials with a 10-min rest period between each trial, and the best score was used as walking speed. Endurance was determined to be the total amount of time, in minutes, that the mouse remained on the enclosed motorized treadmill (FT-200, Chengdu Techman Software Company, Chengdu, China). Physical activity was measured by assessing voluntary distance ran using a running wheel (80820F, Lafayette Instruments, Lafayette, IN, USA). Briefly, mice were individually housed in the wheel running cages for 4 days, and the average distance they ran per day was counted to score physical activity.

### 2.7 Enzyme-linked immunosorbent assay

The serum was separated from the whole blood by centrifugation at 3,000 rpm for 15 min at 4°C and then stored at −80°C for the subsequent biological analysis. Inflammatory cytokines in human serum were quantified using the human inflammation Luminex multi-factor panel (LX-MultiDTH-10, LabEx Co., Shanghai, China) in accordance with the manufacturer’s instructions. The following inflammatory markers were measured: IL-1, IL-6, IL-10, IL-17, TNF-α, and IFN-γ. Gal-3 concentrations in human and mouse serum were quantified using the Human Gal-3 ELISA Kit (DGAL30, R&D Systems, Minneapolis, MN, USA) and the Mouse Gal-3 ELISA Kit (EMLGALS3, Thermo Fisher Scientific Inc., Waltham, MA, USA) according to the manufacturer’s protocols. Inflammatory cytokine levels in the serum of mice were quantified using IL-6 (ab222503, Abcam, Cambridge, UK), IL-10 (SM1000B, R&D Systems), TNF-α (SMTA00B, R&D Systems), and INF-γ (SMIF00, R&D Systems) ELISA kit.

### 2.8 Statistical analysis

For continuous variables, the Shapiro–Wilk test was performed to test the normality, and Levene’s test was conducted to analyze the homogeneity of variance. The variables complied with the normal distribution, and equal variances assumed were analyzed using the one-way analysis of variance (ANOVA) with Tukey’s *post-hoc* test and expressed as mean ± standard deviation (SD). For variables with equal variances not assumed, the Welch one-way ANOVA with the Games–Howell *post-hoc* test was carried out. In addition, the Kruskal–Wallis ANOVA test was used to compare the differences between the groups for variables that are not normally distributed, and the median and interquartile range were presented. The categorical variables were analyzed using Pearson’s chi-square test and were expressed as counts (percentages). Pearson’s correlation analysis was used to assess the correlations between blood galectin-3 levels and baseline characteristics or laboratory indexes. A *p*-value <0.05 was considered statistically significant. Statistical analyses were performed using SPSS version 26.0 (IBM, Armonk, NY, USA). For the animal experiment, data between two groups were statistically analyzed using Student’s t-test through GraphPad Prism (version 8.0, San Diego, CA, USA) software. Results were presented as mean ± SD, and differences were considered statistically significant when *p*-value <0.05.

## 3 Results

### 3.1 Characteristics of patients

A total of 149 patients (68 men and 81 women) were included according to the inclusion and exclusion criteria. The average age of all patients was 72.04 ± 7.05 years, and the average Fried phenotype score was 1.0. Among the patients, 21.48% were frail, 36.91% were pre-frail, and 41.61% were non-frail. The baseline characteristics of participants among the three groups are shown in **Table 1**. The results showed that there were significant differences in BMI (*p* = 0.023), grip strength (*p* < 0.001), walking speed (*p* < 0.001), CCI (*p* = 0.043), SARC-F score (*p* < 0.001), MNA score (*p* = 0.008), and Barthel index (*p* < 0.001) among the groups. According to the *post-hoc* test, the grip strength (*p* = 0.013), MNA score (*p* = 0.022), and Barthel index (*p* < 0.001) in the frail group exhibited a statistical decrease compared to those in the pre-frail group (**Supplementary Table 1**). Participants with pre-frailty had lower grip strength (*p* = 0.013), walking speed (*p* < 0.001), and Barthel index (*p* < 0.001) compared with non-frail elderlies. As a sarcopenia risk scale, SARC-F showed significantly higher scores in frail individuals than pre-frail and non-frail participants (*p* < 0.001) but showed no difference between the pre-frail and non-frail groups (*p* = 0.718). There was no statistical difference among the three groups, including between age, sex, education duration, height, weight, ASM, and RASM. With respect to whole-blood count and biochemical indexes, **Table 2** shows that there were significant differences in BUN and P between the groups (*p* < 0.001). The serum concentrations of BUN and P in the frail group were significantly higher than those in the pre-frail and non-frail groups (*post hoc*, *p* < 0.01, **Supplementary Table 2**).

**Table 1:** Baseline characteristics.

Variables	NF group (n = 62)	PF group (n = 55)	F group (n = 32)	*p*
**Age**, years	70.95 ± 6.65	71.91 ± 6.76	74.38 ± 7.91	0.081^a^
**Sex**, male (%)	27 (43.55)	25 (45.45)	16 (50.00)	0.837^d^
**Education**, years	12.00 (9.00, 16.00)	9.00 (9.00, 16.00)	9.00 (6.00, 15.00)	0.423^c^
**Height**, m	1.60 (1.54, 1.70)	1.60 (1.54, 1.69)	1.63 (1.55, 1.68)	0.628^c^
**Weight**, kg	65.11 ± 11.75	60.44 ± 11.05	61.53 ± 10.04	0.067^a^
**BMI, kg/m^2^**	24.78 ± 3.19	23.34 ± 3.88	23.14 ± 2.96	0.023^b^
**Grip strength**, kg	35.90 (25.18, 40.30)	25.20 (22.30, 36.40)	21.55 (16.58, 25.65)	<0.001^c^
**Walking speeds**, m/s	0.96 (0.86, 1.01)	0.78 (0.73, 0.96)	0.73 (0.65, 0.90)	<0.001^c^
**ASM**, kg	18.60 (15.50, 23.18)	19.20 (13.90, 22.20)	19.60 (16.63, 23.25)	0.663^c^
**RASM**, kg/m^2^	7.65 (6.45, 9.05)	7.27 (5.67, 8.46)	7.60 (6.99, 9.03)	0.158^c^
**CCI**	1.00 (1.00, 2.00)	1.00 (1.00, 2.00)	2.00 (1.00, 4.00)	0.043^c^
**SARC-F score**	0.00 (0.00, 0.00)	0.00 (0.00, 1.00)	1.00 (0.00, 4.00)	<0.001^c^
**Barthel index**	100.00 (100.00, 100.00)	95.00 (90.00, 100.00)	80.00 (65.00, 85.00)	<0.001^c^
**MNA score**	27.50 (25.88, 29.00)	27.50 (25.50, 28.50)	25.75 (24.00, 27.50)	0.008^c^
**FP score**	0.00 (0.00, 0.00)	1.00 (1.00, 2.00)	3.00 (3.00, 4.00)	<0.001^c^

NF group, non-frail group; PF group, pre-frail group; F group, frail group; BMI, body mass index; ASM, appendicular skeletal muscle mass; RASM, relative ASM; CCI, Charlson Comorbidity Index; SARC-F, sarcopenia risk; MNA, Mini-Nutritional Assessment; FP, Fried frailty phenotype.

a One-way ANOVA test, Tukey’s post-hoc test, mean ± SD.

b Welch one-way ANOVA test, Games–Howell post-hoc test, mean ± SD.

c Kruskal–Wallis ANOVA test, Bonferroni post-hoc test, median (interquartile).

d Pearson’s chi-square.

**Table 2:** Hematological and hema-biochemical parameters of participants.

Variables	NF group (n = 62)	PF group (n = 55)	F group (n = 32)	*p*
**Hb**, g/L	143.00 (135.50, 150.75)	141.00 (134.00, 149.00)	140.00 (130.00, 148.25)	0.140^c^
**RBC**, 10^12^/L	4.60 (4.40, 5.07)	4.64 (4.37, 5.03)	4.52 (4.25, 4.86)	0.377^c^
**WBC**, 10^12^/L	6.00 (4.89, 7.09)	5.70 (4.30, 7.00)	5.35 (4.93, 7.18)	0.529^c^
**PLT**, 10^9^/L	208.50 (177.75, 235.50)	200.00 (155.00, 237.00)	208.50 (169.50, 246.00)	0.492^c^
**ALT**, IU/L	21.95 (15.75, 28.90)	22.70 (15.00, 29.20)	23.25 (17.63, 29.30)	0.879^c^
**AST**, IU/L	23.80 (20.88, 30.93)	26.00 (18.00, 30.60)	25.60 (22.03, 28.70)	0.876^c^
**GGT**, IU/L	26.40 (19.00, 34.53)	20.90 (16.00, 27.90)	19.90 (16.43, 39.50)	0.050^c^
**BUN**, mmol/L	5.27 (4.58, 6.15)	5.65 (4.82, 6.50)	7.66 (5.48, 9.20)	<0.001^c^
**Cr**, mmol/L	69.20 (58.50, 81.78)	64.00 (59.20, 74.30)	65.50 (54.05, 81.78)	0.591^c^
**UA**, mmol/L	332.19 ± 70.64	327.83 ± 70.32	326.13 ± 86.52	0.917^a^
**Na**, mmol/L	141.60 (140.00, 143.00)	141.80 (140.30, 143.00)	141.90 (141.30, 143.00)	0.363^c^
**K**, mmol/L	4.36 ± 0.62	4.26 ± 0.53	4.21 ± 0.35	0.311^b^
**Ca**, mmol/L	2.34 (2.28, 2.42)	2.36 (2.28, 2.43)	2.36 (2.30, 2.40)	0.992^c^
**P**, mmol/L	1.13 (1.05, 1.28)	1.13 (1.03, 1.30)	1.32 (1.25, 1.48)	<0.001^c^
**Glu**, mmol/L	5.68 (4.99, 6.96)	5.14 (4.81, 5.96)	5.24 (4.71, 6.24)	0.054^c^
**TC**, mmol/L	4.68 ± 1.02	4.82 ± 0.94	4.63 ± 0.97	0.611^a^
**TG**, mmol/L	1.44 (1.08, 2.19)	1.48 (1.09, 1.86)	1.38 (1.11, 2.03)	0.953^c^

NF group, non-frail group; PF group, pre-frail group; F group, frail group; Hb, hemoglobin; RBC, red blood cell; WBC, white blood cell; PLT, platelet; ALT, alanine aminotransferase; AST, aspartate transaminase; GGT, γ-glutamyl transferase; BUN, blood urea nitrogen; Cr, creatinine; UA, uric acid; TC, total cholesterol; TG, triglycerides.

a One-way ANOVA test, Tukey’s post-hoc test, mean ± SD.

b Welch one-way ANOVA test, Games–Howell post-hoc test, mean ± SD.

c Kruskal–Wallis ANOVA test, Bonferroni post-hoc test, median (interquartile).

### 3.2 Gal-3 whole-blood level depending on the frailty status

**Figure 1:**
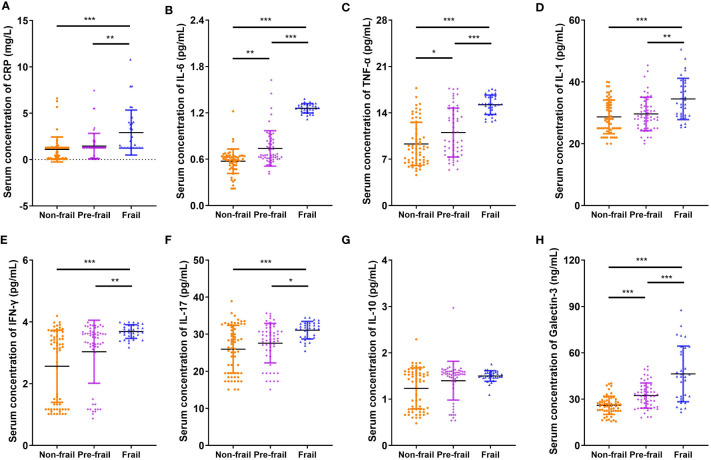
Comparison of inflammatory cytokines and galectin-3. Serum concentration of **(A)** CRP, **(B)** IL-6, **(C)** TNF-α, **(D)** IL-1, **(E)** IFN-γ, **(F)** IL-17, **(G)** IL-10, and **(H)** galectin-3. N = 62 in non-frail group, N = 55 in pre-frail group, and N = 32 in frail group. Data are presented as mean ± SD. *p < 0.05, **p < 0.01, ***p < 0.001, ANOVA with *post-hoc* test. CRP, C-reactive protein.

**Figure 1** shows that the serum Gal-3 contents of frail elderlies (46.34 ± 17.99 mmol/L) were significantly higher than those of pre-frail elderlies (32.30 ± 8.14 mmol/L, *post hoc p* < 0.001) and non-frail elderlies (26.00 ± 5.87 mmol/L, *post hoc p* < 0.001). In the comparison of inflammatory factors in the three groups, serum CRP, IL-1, IL-6, IL-17, TNF-α, and IFN-γ were significantly increased in the frail group than the other two groups (*post hoc p* < 0.05). We did not find any significant difference in IL-10 levels among the three groups (ANOVA *p* = 0.111). Notably, IL-10 and IL-1 serum levels showed statistically significant differences in men but no significant differences in women (**Supplementary Table 3**). As shown in **Table 3**, negative correlations were found between blood Gal-3 and grip strength (*p* = 0.005), Barthel index (*p* < 0.001), and MNA (*p* = 0.041). In addition, positive correlations between blood Gal-3 and pro-inflammatory cytokine IL-6, IL-17, TNF-α, and IFN-γ were identified. Notably, blood Gal-3 showed a strong positive correlation with FP scores (correlation coefficient R = 0.638, *p* < 0.001). The results suggested that blood Gal-3 level was closely connected with inflammation and frail status in elderly participants.

**Table 3:** The correlation test with Pearson’s coefficient, according to the blood galectin-3 levels, baseline characteristics, and laboratory indexes.

Variables	Correlation R	*p*-Value	Variables	Correlation R	*p*-Value
Grip strength	−0.231	0.005	P	0.198	0.016
SARC-F score	0.407	<0.001	CRP	0.340	<0.001
Barthel index	−0.440	<0.001	IL-6	0.475	<0.001
MNA score	−0.168	0.041	TNF-α	0.389	<0.001
FP score	0.638	<0.001	IFN-γ	0.254	0.002
BUN	0.294	<0.001	IL-17	0.234	0.004

SARC-F, sarcopenia risk; MNA, Mini-Nutritional Assessment; FP, Fried frailty phenotype; CRP, C-reactive protein; BUN, blood urea nitrogen.

### 3.3 Identification of differentially expressed genes

**Figure 2:**
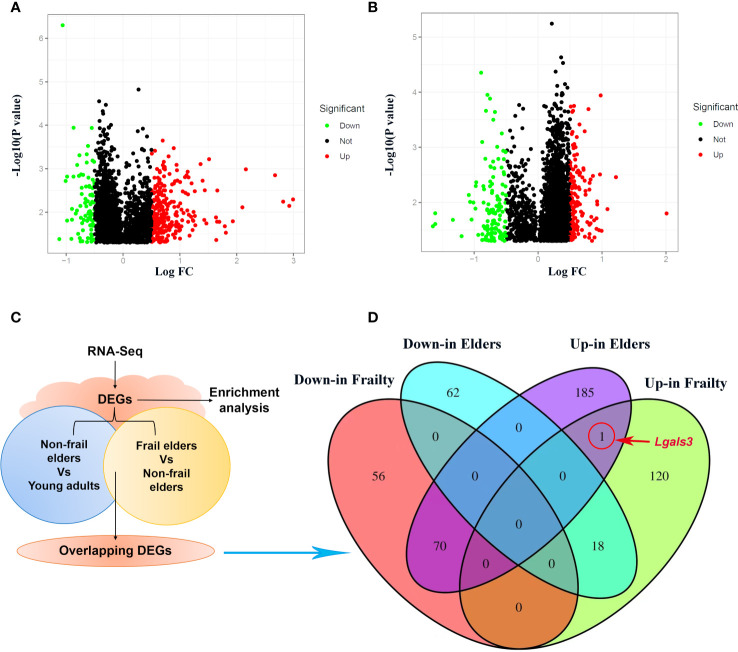
Identification of DEGs in PBMCs with the cutoff criteria of *p* < 0.05. Respective volcano plots of the old *vs.* young group **(A)** and the frail *vs.* non-frail group. **(B)** Red and green plots represent up- and downregulated genes, respectively (|log2 FC| > 0.5 and *p* < 0.05). Black plots represent the remaining genes with no significant difference. **(C)** Scheme of the experimental procedure. **(D)** Venn diagrams representing commonly changed DEGs in the three groups. DEGs differentially expressed genes; FC, fold change; PBMCs, peripheral blood mononuclear cells.

We obtained the gene expression data of PBMC samples from young adults, non-frail elderlies, and frail elderlies (N = 6) and identified the DEGs using the Limma package. Upon setting the cutoff criterion as genes with |log2 fold change (FC)| > 0.5 and *p* < 0.05, we identified 264 DEGs (139 upregulated and 126 downregulated) in the frailty set (frail *vs.* non-frail elderlies) and 335 DEGs (256 upregulated and 80 downregulated) in the aging set (non-frail elderlies *vs.* young adults) as shown by volcano plots (**Figures 2A, B**). Following the experiment in **Figure 2C**, the overlapping DEGs between the aging and frailty sets were further identified. Venn diagram made the visualization of all 89 overlapping DEGs (**Figure 2D**), including one upregulated and zero downregulated in both the frailty and aging sets, 70 upregulated in the aging set but downregulated in the frailty set, and 18 upregulated in the frailty set but downregulated in the aging set (**Supplementary Table 4**). Notably, the only commonly upregulated gene is *LGALS3*, which is the protein-coding gene of Gal-3 (**Figure 2D**). These findings indicated that Gal-3 participates in the regulation of aging and frailty.

### 3.4 GO enrichment analysis and KEGG pathway enrichment of DEGs

**Figure 3:**
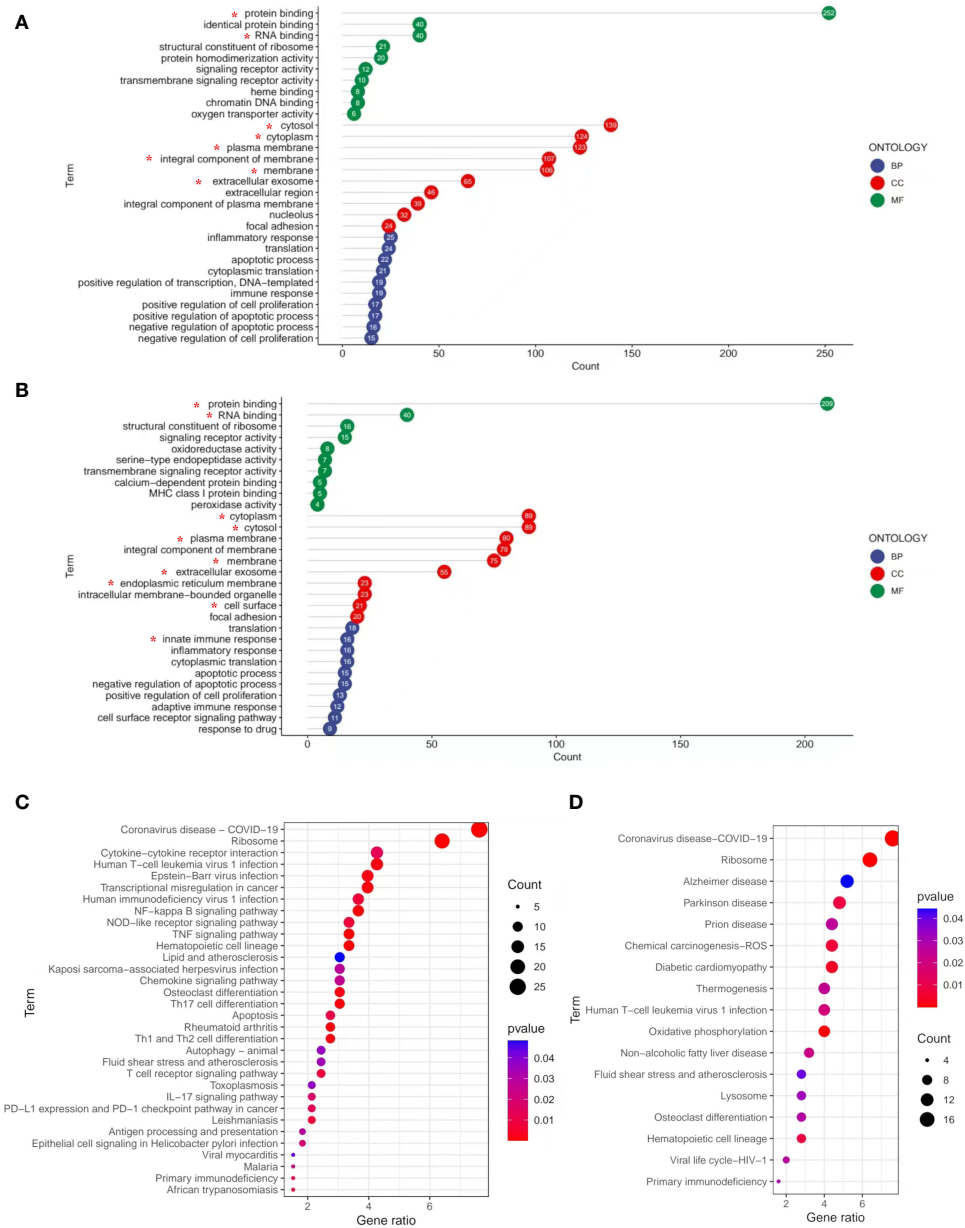
GO and KEGG pathways enrichment analysis of DEGs with the cutoff criteria of *p* < 0.05. **(A)** DEGs in three parts of GO enrichment (BP, CC, and MF) from non-frail elderlies *vs.* young adults. **(B)** DEGs in three parts of GO enrichment (BP, CC, and MF) from frail elderlies *vs.* non-frail elderlies. The number in bubble represents the gene number. * Indicates that the *LGALS3* was in this module. Bubble maps show the top 30 KEGG pathways associated with DEGs of non-frail elderlies *vs.* young adults **(C)** and frail elderlies *vs.* non-frail elderlies **(D)**. Significantly enriched pathways are indicated in y-axis. Gene ratio in the x-axis represents the enrichment levels. Color of the dot stands for the different *p*-values, and the size of the dot reflects the number of target genes enriched in the corresponding pathway. BP, biological process; CC, cellular component; DEG, differentially expressed gene; GO, Gene Ontology; MF, molecular function; KEGG, Kyoto Encyclopedia of Genes and Genomes.

To better determine the role of DEGs in the pathological process of frailty, the DEGs between the young, non-frail, and frail groups were input to DAVID 6.8 for GO functional analysis to understand the functional distribution characteristics. The DEGs were examined, and based on the *p* < 0.05, the functions of DEGs were mainly enriched and described by lollipop plots as the top 10 GO sub-ontologies as follows: BP, MF, and CC. As can be seen (**Figures 3A, B**), several similar GO terms are shown in the frailty and aging sets. The top 3 significant terms from the analysis showed that in the MF category, the diverse DEGs were involved in protein binding, RNA binding, and structural constituent of ribosome; *LGALS3* participated in the first two. For the CC category, the different mRNAs were correlated with cytosol/cytoplasm, plasma membrane/integral component of the plasma membrane, and extracellular exosome, all of them including *LGALS3*. For the BP category, the diverse DEGs were mainly enriched in inflammatory response, translation, innate immune response, and apoptotic process. Meanwhile, *LGALS3* was enriched in the innate immune response. KEGG analyses were presented in dot plots based on the cutoff threshold *p*-value <0.05 (**Figures 3C, D**). In both older and frail cases, coronavirus disease–COVID-19 and ribosome exhibited respectively the first and second significantly ranked gene annotations in KEGG enrichment. However, there are still some differences between the aging and frailty sets. The most significant pathways in the aging set were identified to be cytokine–cytokine receptor interaction, Epstein–Barr virus infection, transcriptional misregulation in cancer, NF-kappa B signaling pathway, and TNF signaling pathway (**Figure 3C**). Enrichment of frailty-related DEGs was mostly in Alzheimer’s disease (AD), Parkinson’s disease, prion diseases, chemical carcinogenesis, and diabetic cardiomyopathy (**Figure 3D**).

### 3.5 Animal experiment

**Figure 4:**
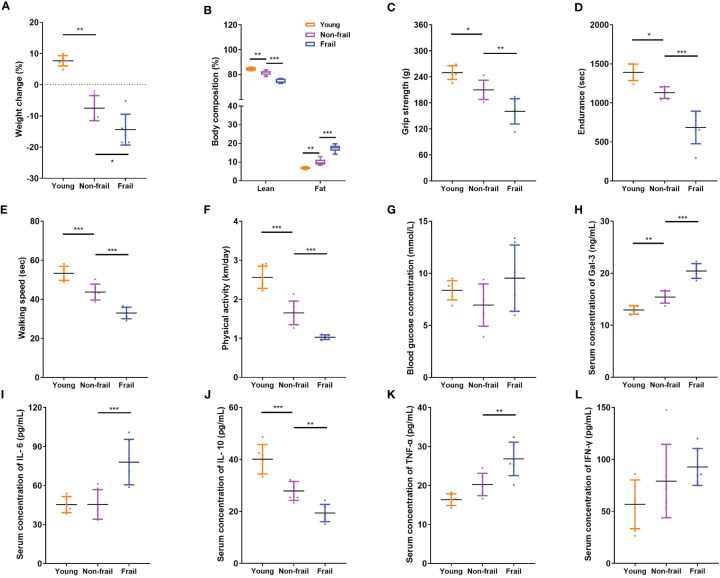
Body composition, physical function, and serum inflammatory cytokines levels in mice. **(A)** Weight changes of mice in 1 month. **(B)** Body composition. **(C)** Grip strength. **(D)** Endurance. **(E)** Walking speed. **(F)** Physical activity per day. **(G)** Fasting blood glucose concentration. **(H)** Serum Gal-3 levels. **(I)** Serum IL-6 levels. **(J)** Serum IL-10 levels. **(K)** Serum TNF-α levels. **(L)** Serum IFN-γ levels. N = 6 per group. Data are presented as mean ± SD. *p < 0.05, **p < 0.01, ***p < 0.001, ANOVA with *post-hoc* test.

Old (23 months) mice were identified to have a positive marker of frailty using the cutoff values mentioned in the previous study (32), including walking speed (38.0 s), strength (220.3 g), endurance (944.2 s), and physical activity (1.088 km per day). For body weight, the mice were positive for the frailty marker if they lost 10% weight or greater during 1 month. Old non-frail mice were lighter (*p* < 0.01) and possessed more body fat (*p* < 0.01) than young mice (**Figures 4A, B**). The same changes were observed between frail and non-frail mice (*p* < 0.05, **Figures 4A, B**). As with body weight and body lean percentage loss, physical performance such as grip strength (**Figure 4C**), time to fatigue (i.e., endurance, **Figure 4D**), and time on the rotarod (i.e., walking speed, **Figure 4E**) were less (*p* < 0.05) for frail mice compared to non-frail mice. Physical activity measured by daily voluntary wheel run distance was 60.3% greater in the non-frail group compared to the frail group (*p* < 0.001, **Figure 4F**). Also, the serum Gal-3 levels of mice in the young group and the non-frail group were significantly lower than those in the frail group (*p* < 0.0001, **Figure 4H**). As for inflammation, the serum levels of IL-6 and TNF-α of mice in the frail group were significantly increased than those of the non-frail group (*p* < 0.01, **Figures 4I, K**) but showed no statistical difference between the non-frail group and young group (*p* > 0.05, **Figures 4I, K**). Meanwhile, the IL-10 concentration in the frail group was significantly decreased (*p* < 0.01, **Figure 4J**). There was no statistical difference in the blood levels of the fasting glucose and IFN-γ levels of mice among the three groups (*p* > 0.05, **Figures 4G, L**).

## 4 Discussion

Currently, the aggressive comorbidities and adverse events associated with frail older adults pose a great challenge in the identification of better and easier biomarkers. Previous studies indicated that selected metabolic, hematologic, and inflammatory biomarkers are significantly associated with frailty in older adults (34). In this work, we aimed to explore the circulating factors in the blood of non-frail and frail older adults to identify the potential biomarkers or small-molecule mediators of frailty. The key findings were as follows: 1) the blood Gal-3 level was significantly elevated in the frail group compared to the non-frail group, and both humans and mice showed the same trend; 2) significant positive correlations between serum Gal-3 level and inflammatory cytokines CRP, IL-6, IL-17, TNF-α, and IFN-γ were observed, along with a negative correlation with grip strength, Barthel index, and MNA score; 3) *LGALS3*, the protein-coding gene of Gal-3, was identified as the commonly un-regulated gene in frailty-related DEGs and aging-related DEGs, related to protein binding, RNA binding, and the biological process of innate immune response.

Frailty is closely related to aging. They share a cassette of similar phenotypes, such as physical function decline, immune defense reduction, metabolic disturbance, and nutritional imbalance (35). Although the association between frailty and aging has long been documented, the mechanisms by which it occurs remain unclear. This study showed that frailty and aging may share a common signal transduction pathway and mechanism of chronic inflammation. In the current research, a total of 139 up- and 126 downregulated DEGs in the frailty set, along with 256 up- and 80 downregulated DEGs in the aging set were identified, and the subsequent analysis was carried out on them (**Figures 2A, B**). Several common terms were found in frailty and aging set *via* gene annotations of DEGs. Both of them were related to the regulations of biological processes such as inflammatory response, translation, immune response, cell proliferation, and apoptotic process. Co-regulated molecular functions were a structural constituent of ribosome, protein binding, RNA binding ability, and transmembrane signaling receptor activity. Cellular components like cytoplasm, plasma membrane, and extracellular exosome were found at higher levels (**Figures 3A, B**). KEGG pathways enrichment analysis disclosed that coronavirus disease–COVID-19, ribosome, human T-cell leukemia virus 1 infection, hematopoietic cell lineage, and primary immunodeficiency were shared in frailty and aging (**Figures 3C, D**). Most of these pathways were involved in virus infection and immunodeficiency, which consisted of the “inflamm-aging” concept in previous studies (36). Inflamm-aging, often referred to as a chronic low-grade inflammatory state in aging, would mobilize local inflammatory cells to secrete several cytokines in response to sustained stimuli (25). Interestingly, *LGALS3* was upregulated in both the aging and frailty sets (**Figure 2D**) and enriched in most of these overlapping GO terms above. Also, high serum Gal-3 levels in old mice and adults with frailty were observed, along with a positive correlation with FP scores and some inflammatory factors. In addition, the age difference of Gal-3 has been also observed in mice (**Figure 4H**). Collectively, these findings suggest that peripheral Gal-3 is related to age-associated frailty and inflammation.

Elevated levels of Gal-3 regarded as a marker of many chronic diseases, such as heart failure, neurodegenerative disorders, and metabolic diseases, have been widely recognized (37). Gal-3 was found to be causally involved in cardiac adverse remodeling, inflammation, and failure by affecting the functions of cardiac fibroblasts and macrophages. It promoted pro-fibrotic responses while at the same time inhibiting the repair of the damaged myocardium (38). However, Lgals3^−/−^ mice demonstrated less home cage movement and more perturbation of behavioral circadian rhythms as compared to wild-type controls (39). For these new traits, further studies examining the consequences of Lgals3 loss at synaptic, neuronal, ensemble, and tissue levels of organization are required to determine the precise underlying mechanisms. Recently, Gal-3 has been implicated in the development of metabolic disorders because it plays a role in glucose homeostasis, adipocyte differentiation, insulin resistance, and diabetes complications (40–42). As to the neuroinflammation and neurodegeneration in the brain, previous studies mentioned that Gal-3 stands out as a key pathological biomarker of AD pathology (43, 44), which was consistent with our KEGG analysis results (**Figure 3D**). Gal-3 is considered a pivotal tuner of macrophage and microglial activities, and its overexpression by microglia/macrophages may be damaging for lesions within the aging neural system (45, 46). Gal-3 induces an anti-inflammatory microglial phenotype through the IL-4 receptor pathway (47, 48), whereas the interaction of Gal-3 with TLR4 receptors in the acute phase of neuroinflammation exacerbates neural cell death and prolongs inflammation (49). Gal-3 also augments monocyte–monocyte interactions that lead to alternative macrophage activation (M2 phenotype), chronic inflammatory response, and fibrotic diseases (50). Therefore, Gal-3 can be viewed as a regulatory molecule acting at various stages along the continuum from acute inflammation to chronic inflammation. In the context of chronic inflammation, local immune cells release inflammatory cytokines as part of the inflammatory secretome, including IL-1, IL-6, TNF-α, IL-17, and granulocyte colony-stimulating factor (G-CSF) (36). Recently, a meta-analysis including 53 cross-sectional studies represented that there were higher CRP, IL-6, and TNF-α levels in older adults with frailty compared with the control group, consistent with our results (51). From this perspective, the elevated serum levels of inflammatory factors CRP, IL-6, IL-1, IL-17, TNF-α, and IFN-γ may reflect an endophenotypic expression of inflamm-aging in elderly participants with frailty (**Figure 1**). However, there was no significant difference in anti-inflammatory factor IL-10 levels among frailty, pre-frailty, and non-frailty in elderly adults. Accordingly, we found that circulating Gal-3 was positively correlated with pro-inflammatory factors CRP, IL-6, IL-17, TNF-α, and IFN-γ (**Table 3**). It is widely known that Gal-3 promoted IL-4-induced M2 macrophage activation and profibrotic process (52). However, recent research showed that inhibition of Gal-3 with GB0139 reduced the predominance of the pro-inflammatory M1 phenotype, along with the reduction of LPS-mediated increases in IL-6, TNFα, and macrophage inflammatory protein-1-alpha (MIP-1) (53). Hence, Gal-3 may adopt a pro-inflammatory role in response to M1 macrophage stimuli. Based on our findings, we propose that Gal-3 plays an important role in the pro-inflammatory process of age-associated frailty. The upregulated expression of Gal-3 in PBMC is one of the contexts of immune response biology that may merit thorough investigation in follow-up studies.

There were several limitations to this study. First, our present study analyzed the cross-sectional association between frailty and blood Gal-3 concentrations. Hence, no causation between these two could be established from our study. The cause-and-effect relationship between frailty and circulating Gal-3 contents needs to be verified further. Second, the association of Gal-3 with frailty might vary across organs and tissues (37). We only tested the serum Gal-3 level in whole-blood samples and gene expression level in human PBMCs so that it could reflect the association of frailty and Gal-3 contents in human and mouse whole blood. Further studies should be conducted to explore the association between Gal-3 and frailty in other tissues or organs. Although in this study blood Gal-3 had a positive correlation with inflammatory cytokines CRP, IL-6, TNF-α, etc., in old adults with frailty, its impact on frailty and the mechanism behind is still unclear. Deeper functional experiments should be taken into account in further research. Finally, the amount of experimental data is limited. In the analysis of some inflammatory factors whose individual variability is large, we should mention that we could not exclude the possibility of small-study effects due to the small number of studies included.

In conclusion, the present study demonstrated that high blood Gal-3 seems to be a risk factor for frailty and is positively associated with inflammatory cytokines. Identification of novel markers for diagnosis and potential targets for the treatment of frailty is an active area of aging-related disease research. Few studies have investigated the function and changes of serum Gal-3 in elderly individuals with frailty. Our findings uncover the association of blood Gal-3 and frailty, provide new insight into the pathogenesis of frailty, and explore the manipulation of Gal-3 expression as a potentially novel therapeutic strategy in frailty.

## Data availability statement

The datasets presented in this study can be found in online repositories. The names of the repository/repositories and accession number(s) can be found in the article/**Supplementary Material**.

## Ethics statement

The animal study was reviewed and approved by the research ethics committee of the School of Life Science of Fudan University.

## Author contributions

XJ, ZJ, and YQ: design, acquisition, analysis, and interpretation of data and drafted the manuscript. JY and YZ: acquisition and analysis of study data and revised the manuscript. JW and BY: analysis and interpretation of data and revised the manuscript. YXH and WG: design, interpretation of data, and revised the manuscript. YQH, JC, and ZB: conception, design, acquisition, analysis, and interpretation of data and revised the manuscript. All authors agreed to be accountable for all aspects of the work and approved the final manuscript.
