# Supplementary material for: High blood galectin-3 level associated with risk of frailty in aging
Source: Front Endocrinol (Lausanne). 2023 Sep 25;14:1189192. doi: 10.3389/fendo.2023.1189192 (PMC10560881; doi:10.3389/fendo.2023.1189192)
Supplement: Supplementary file 1 [file Table_1.docx]

**Supplementary Table 1. ANOVA with post hoc test of baseline characteristics**

| **Variables** | **Statistic value** | ***P*** | **Post hoc *P* value** | | |
| --- | --- | --- | --- | --- | --- |
|  |  |  | NF/PF | NF/F | PF/F |
| **Age**, years | 2.558 | 0.081^a^ |  |  |  |
| **Sex**, male (%) | 0.355 | 0.837^d^ |  |  |  |
| **Education**, years | 1.721 | 0.423^c^ |  |  |  |
| **Height**, m | 0.932 | 0.628^c^ |  |  |  |
| **Weight**, kg | 2.752 | 0.067^a^ |  |  |  |
| **BMI, kg/m^2^** | 3.950 | 0.023^b^ | 0.080 | 0.041 | 0.961 |
| **Grip strength**, kg | 29.173 | <0.001^c^ | 0.013 | <0.001 | 0.013 |
| **Walking speeds**, m/s | 36.935 | <0.001^c^ | <0.001 | <0.001 | 0.222 |
| **ASM**, kg | 0.823 | 0.663^c^ |  |  |  |
| **RASM**, kg/m^2^ | 3.693 | 0.158^c^ |  |  |  |
| **CCI** | 6.283 | 0.043^c^ | 0.777 | 0.037 | 0.393 |
| **SARC-F score** | 26.009 | <0.001^c^ | 0.718 | <0.001 | <0.001 |
| **Barthel Index** | 70.935 | <0.001^c^ | <0.001 | <0.001 | <0.001 |
| **MNA score** | 9.715 | 0.008^c^ | 1.000 | 0.010 | 0.022 |
| **FP score** | 140.976 | <0.001^c^ | <0.001 | <0.001 | <0.001 |

Note：^a^ One-way ANOVA test, Tukey post hoc test, mean ± SD; ^b^ Welch one-way ANOVA test, Games-Howell post hoc test, mean ± SD; ^c^ Kruskal-Wallis ANOVA test, Bonferroni post hoc test, median (interquartile); ^d^ Pearson Chi-Square. NF group, non-frail group; PF group, pre-frail group; F group, frail groupl; BMI, body mass index; ASM, appendicular skeletal muscle mass; RASM, relative ASM; CCI, Charlson Comorbidity Index; SARC-F, sarcopenia risk; MNA, Mini-Nutritional Assessment; FP, Fried frailty phenotype.

**Supplementary Table 2. ANOVA with post hoc test of hematological and hema-biochemical parameters**

| **Variables** | **Statistic value** | ***P*** | **Post Hoc *P* value** | | |
| --- | --- | --- | --- | --- | --- |
|  |  |  | NF/PF | NF/F | PF/F |
| **Hb**, g/L | 3.933 | 0.140^c^ |  |  |  |
| **RBC**, 10^^12^/L | 1.949 | 0.377^c^ |  |  |  |
| **WBC**, 10^^12^/L | 1.274 | 0.529^c^ |  |  |  |
| **PLT**, 10^^9^/L | 1.420 | 0.492^c^ |  |  |  |
| **ALT**, IU/L | 0.258 | 0.879^c^ |  |  |  |
| **AST**, IU/L | 0.266 | 0.876^c^ |  |  |  |
| **GGT**, IU/L | 5.972 | 0.050^c^ |  |  |  |
| **BUN**, mmol/L | 21.517 | <0.001^c^ | 0.640 | <0.001 | 0.002 |
| **Cr**, mmol/L | 1.051 | 0.591^c^ |  |  |  |
| **UA**, mmol/L | 0.087 | 0.917^a^ |  |  |  |
| **Na**, mmol/L | 2.029 | 0.363^c^ |  |  |  |
| **K**, mmol/L | 1.183 | 0.311^b^ |  |  |  |
| **Ca**, mmol/L | 0.017 | 0.992^c^ |  |  |  |
| **P**, mmol/L | 19.772 | <0.001^c^ | 1.000 | <0.001 | <0.001 |
| **Glu**, mmol/L | 5.849 | 0.054^c^ |  |  |  |
| **TC**, mmol/L | 0.495 | 0.611^a^ |  |  |  |
| **TG**, mmol/L | 0.096 | 0.953^c^ |  |  |  |

Note：^a^ One-wayANOVA test, Tukey post hoc test, mean ± SD; ^b^ Welch one-way ANOVA test, Games-Howell post hoc test, mean ± SD; ^c^ Kruskal-Wallis ANOVA test, Bonferroni post hoc test, median (interquartile). NF group, non-frail group; PF group, pre-frail group; F group, frail groupl.

**Supplementary Table 3. Comparison of Galectin-3 and inflammatory cytokines stratified by sex.**

| **Variables** | **Sex** | **NF group (M=27; F=35)** | **PF group (M=25; F=30)** | **F group (M=16; F=16)** | ***P*** |
| --- | --- | --- | --- | --- | --- |
|  |  |  |  |  |  |
| **CRP**, mg/L | Male | 0.27 (0.08, 1.28) | 1.28 (0.85, 1.28) | 1.28 (1.28, 4.38) * | <0.001^b^ |
|  | Female | 1.28 (0.38, 1.35) | 1.28 (1.28, 1.73) | 1.58 (1.28, 4.00) * | 0.005^b^ |
| **IL-6**, pg/ml | Male | 0.59 (0.47, 0.68) | 0.63 (0.61, 0.81) | 1.25 (1.20, 1.31) * # | <0.001^b^ |
|  | Female | 0.60 (0.55, 0.64) | 0.65 (0.61, 0.86) † | 1.27 (1.23, 1.30) * # | <0.001^b^ |
| **TNF-α**, pg/ml | Male | 9.57 ± 3.42 | 11.13 ± 3.39 | 15.23 ± 1.41 * # | <0.001^a^ |
|  | Female | 8.58 (6.74, 10.01) | 9.35 (7.56, 14.63) | 15.32 (14.07, 16.45) * # | <0.001^b^ |
| **IL-1α**, pg/ml | Male | 25.00 (24.00, 33.34) | 30.78 (27.34, 34.60) | 35.89 (31.87, 41.92) * | 0.001^b^ |
|  | Female | 29.08 ± 5.06 | 28.59 ± 4.00 | 32.17 ± 5.31 | 0.073^a^ |
| **IFN-γ**, pg/ml | Male | 1.50 (1.17, 3.34) | 3.57 (2.22, 3.76) | 3.78 (3.56, 3.93) * | <0.001^b^ |
|  | Female | 3.31 (1.33, 3.70) | 3.41 (3.15, 3.64) | 3.66 (3.44, 3.79) * | 0.022^b^ |
| **IL-17**, pg/ml | Male | 24.00 (18.40, 29.52) | 28.70 (24.13, 32.28) | 31.20 (28.91, 32.99) * | 0.005^b^ |
|  | Female | 28.33 (21.70, 32.57) | 28.56 (25.67, 30.67) | 32.17 (29.85, 32.92) * # | 0.016^b^ |
| **IL-10**, pg/ml | Male | 0.97 (0.72, 1.51) | 1.53 (1.28, 1.62) | 1.51 (1.47, 1.57) * | 0.020^b^ |
|  | Female | 1.52 (0.78, 1.64) | 1.54 (1.42, 1.59) | 1.49 (1.41, 1.58) | 0.889^b^ |
| **Galectin-3**, ng/mL | Male | 25.93 ± 5.37 | 33.80 ± 8.51 † | 44.86 ± 19.47 * | <0.001^a^ |
|  | Female | 26.06 ± 6.30 | 31.06 ± 7.74 † | 47.81 ± 16.89 * # | <0.001^a^ |

Note: ^a^ Welch one-way ANOVA test, Games-Howell post hoc test, mean ± SD; ^b^ Kruskal-Wallis ANOVA test, Bonferroni post hoc test, median (interquartile). NF group, non-frail group; PF group, pre-frail group; F group, frail groupl. † indicates *P* < 0.05 NF group vs. PF group; * indicates *P* < 0.05 NF group vs. F group; # indicates *P* < 0.05 PF group vs. F group.

**Supplementary Table 4. Identification of the overlapping DEGs.**

|  | **Frailty set**  **(Frail vs non-frail elderly adults)** | | **Aging set**  **(Non-frail elders vs young adults)** | |
| --- | --- | --- | --- | --- |
| **Gene name** | **logFC** | ***P* value** | **logFC** | ***P* value** |
| *AATK* | -0.62507 | 0.014016 | 0.956236 | 0.001874 |
| *AC099489.1* | -0.79244 | 0.026686 | 1.152739 | 0.001581 |
| *ACSL1* | -0.65463 | 0.030113 | 0.746013 | 0.020308 |
| *ADARB2* | -0.84449 | 0.005774 | 0.69993 | 0.027114 |
| *ADGRG3* | -1.3327 | 0.020671 | 2.161166 | 0.001033 |
| *ALOX5* | -0.50903 | 0.005114 | 0.571506 | 0.008306 |
| *AMPD2* | -0.8522 | 0.01005 | 1.117728 | 0.001422 |
| *ANXA3* | -0.70517 | 0.021558 | 0.974455 | 0.019262 |
| *ARPP19* | 0.769885 | 0.022685 | -0.83221 | 0.010783 |
| *ATG2A* | -1.08324 | 0.00733 | 1.143663 | 0.003889 |
| *BCL6* | -0.89063 | 4.42E-05 | 0.716245 | 0.00098 |
| *CA4* | -0.79483 | 0.013312 | 1.137299 | 0.001175 |
| *CCNJL* | -0.68319 | 0.03004 | 0.858595 | 0.01694 |
| *CD3D* | 0.608812 | 0.002833 | -0.53563 | 0.004171 |
| *CD82* | -0.57452 | 0.040012 | 0.591407 | 0.041732 |
| *CD8A* | 0.968807 | 0.003103 | -0.63314 | 0.048537 |
| *CD8B* | 0.655421 | 0.010778 | -0.81097 | 0.009177 |
| *CHD4* | -0.63848 | 0.007716 | 0.628966 | 0.005281 |
| *CIAO2A* | 0.729511 | 0.000512 | -0.56128 | 0.032107 |
| *CSF3R* | -0.78194 | 0.037282 | 1.130738 | 0.009126 |
| *CYP4F3* | -0.87113 | 0.037751 | 1.437943 | 0.003138 |
| *DDIT3* | -0.69516 | 0.015503 | 0.599507 | 0.014947 |
| *DENND3* | -0.58364 | 0.035999 | 0.894132 | 0.002196 |
| *DHX34* | -0.68351 | 0.002389 | 0.892091 | 0.000337 |
| *DYSF* | -0.97489 | 0.005035 | 0.96958 | 0.007379 |
| *ECE1* | -0.71877 | 0.004613 | 0.739267 | 0.003162 |
| *EEF1A1* | 0.573638 | 0.011955 | -0.61962 | 0.039526 |
| *ELL* | -0.73998 | 0.00227 | 0.94848 | 0.001086 |
| *FBXL13* | -0.59335 | 0.034635 | 0.634615 | 0.027925 |
| *FOS* | -1.19623 | 0.040931 | 0.925787 | 0.01167 |
| *GLA* | -0.76605 | 0.015727 | 0.843945 | 0.003292 |
| *GMPR* | -0.66628 | 0.023913 | 0.510367 | 0.012548 |
| *GZMK* | 0.923718 | 0.009558 | -1.0104 | 0.001917 |
| *HBA1* | -1.64633 | 0.027049 | 2.992291 | 0.005091 |
| *HBA2* | -1.60831 | 0.024595 | 2.818236 | 0.005723 |
| *HIST1H4K* | 0.647866 | 0.005758 | -0.5055 | 0.002445 |
| *HLA-DPA1* | 0.688841 | 0.0173 | -0.79054 | 0.031495 |
| *ICAM1* | -0.66238 | 0.029016 | 0.74145 | 0.011092 |
| *ITGAX* | -0.62556 | 0.024967 | 0.853805 | 0.005744 |
| *KDM6B* | -0.70494 | 0.013479 | 0.716405 | 0.003135 |
| *KLRG1* | 0.954875 | 0.031812 | -0.98482 | 0.015614 |
| *LGALS3* | 0.788461 | 0.000203 | 0.697812 | 0.000225 |
| *LILRB3* | -0.70301 | 0.001809 | 0.557126 | 0.027286 |
| *LIMK2* | -1.06311 | 0.009768 | 1.363621 | 0.000784 |
| *MBOAT2* | -0.87387 | 0.000801 | 0.720931 | 0.001495 |
| *MBOAT7* | -0.79577 | 0.013831 | 1.050731 | 0.00333 |
| *MED25* | -0.52697 | 0.049725 | 0.591039 | 0.023669 |
| *MMP25* | -1.04 | 0.020462 | 1.658423 | 0.00317 |
| *MPP1* | -0.72998 | 0.039997 | 0.579053 | 0.021382 |
| *NACA* | 0.926426 | 0.010882 | -0.71649 | 0.046638 |
| *NFAM1* | -0.59501 | 0.017172 | 0.522614 | 0.043624 |
| *NUAK2* | -0.81473 | 0.000218 | 0.733177 | 0.000915 |
| *PACSIN2* | -0.65125 | 0.011583 | 0.677107 | 0.007294 |
| *PDLIM7* | -0.74498 | 0.002215 | 0.863595 | 0.002293 |
| *PFKFB3* | -0.96721 | 0.004302 | 0.923644 | 0.011144 |
| *PHACTR1* | -0.72464 | 0.043414 | 0.72609 | 0.012073 |
| *PHOSPHO1* | -0.59717 | 0.041696 | 0.902356 | 0.007582 |
| *PLAUR* | -0.77716 | 0.01078 | 1.004715 | 0.002028 |
| *PLIN5* | -0.51311 | 0.022125 | 0.706493 | 0.002444 |
| *PLK3* | -0.69243 | 0.04446 | 0.948108 | 0.012071 |
| *PROK2* | -0.85119 | 0.02691 | 0.988133 | 0.026643 |
| *RAB11FIP1* | -0.62126 | 0.001622 | 0.716926 | 0.000694 |
| *RFX2* | -0.60339 | 0.010615 | 0.669746 | 0.007718 |
| *RNF10* | -0.50263 | 0.007417 | 0.504053 | 0.010737 |
| *RNF122* | -0.5558 | 0.004729 | 0.55428 | 0.012531 |
| *RPL23* | 0.581463 | 0.001668 | -0.77707 | 0.001463 |
| *RPL34* | 0.605605 | 0.00065 | -0.76794 | 0.000491 |
| *RPS12* | 0.883232 | 0.043077 | -1.12194 | 0.04159 |
| *RPS15A* | 0.565582 | 0.001398 | -0.73914 | 0.001353 |
| *RPS17* | 0.555266 | 0.001035 | -0.57828 | 0.004027 |
| *RPS2* | 0.979762 | 0.000114 | -0.6319 | 0.015835 |
| *RPS3A* | 0.729664 | 0.004027 | -0.66369 | 0.012118 |
| *SBNO2* | -0.55461 | 0.00693 | 0.729407 | 0.002472 |
| *SEC14L1* | -0.68873 | 0.021182 | 0.688221 | 0.014215 |
| *SH3TC2* | -0.74837 | 0.004171 | 0.517751 | 0.015153 |
| *SLC11A1* | -0.6013 | 0.008947 | 0.693803 | 0.00758 |
| *SLC16A3* | -0.50336 | 0.040889 | 0.893981 | 0.00578 |
| *SLC19A1* | -0.52534 | 0.027936 | 0.722446 | 0.004099 |
| *SLC25A37* | -0.97963 | 0.012933 | 1.51366 | 0.000604 |
| *SNCA* | -0.75119 | 0.000131 | 0.658443 | 0.013752 |
| *STRADB* | -0.73905 | 0.036282 | 0.941798 | 0.009941 |
| *STX3* | -0.75096 | 0.005084 | 0.726456 | 0.016225 |
| *TLE3* | -0.56727 | 0.00056 | 0.648192 | 0.001206 |
| *TMEM140* | -0.79143 | 0.000112 | 0.614258 | 0.002286 |
| *TREML2* | -0.62064 | 0.011989 | 0.625796 | 0.019358 |
| *TRIM58* | -0.85583 | 0.005055 | 0.689447 | 0.004077 |
| *UBN1* | -0.50987 | 0.010138 | 0.604865 | 0.00334 |
| *XLOC_254035* | -0.72245 | 0.029436 | 0.702226 | 0.021741 |
| *XPO6* | -0.55964 | 0.038018 | 0.77485 | 0.008652 |

Abbreviations: DEGs, differentially expressed genes; FC, fold change.
